# Pregestational Diabetes Mellitus and Adverse Perinatal Outcomes: A Systematic Review and Meta-Analysis

**DOI:** 10.3390/jcm14134789

**Published:** 2025-07-07

**Authors:** Dionysios Gazis, Antigoni Tranidou, Antonios Siargkas, Aikaterini Apostolopoulou, Georgia Koutsouki, Dimitrios G. Goulis, Christos Tsakalidis, Ioannis Tsakiridis, Themistoklis Dagklis

**Affiliations:** 1 3rd Department of Obstetrics and Gynecology, School of Medicine, Faculty of Health Sciences, Aristotle University of Thessaloniki, 54124 Thessaloniki, Greece; 2 Laboratory of Hygiene, Social and Preventive Medicine and Medical Statistics, School of Medicine, Faculty of Health Sciences, Aristotle University of Thessaloniki, 54124 Thessaloniki, Greece; 3 Unit of Reproductive Endocrinology, 1st Department of Obstetrics and Gynecology, School of Medicine, Faculty of Health Sciences, Aristotle University of Thessaloniki, 54124 Thessaloniki, Greece; 4 2nd Neonatal Department and NICU, School of Medicine, Faculty of Health Sciences, Aristotle University of Thessaloniki, 54124 Thessaloniki, Greece

**Keywords:** adverse outcomes, complications, maternal, neonatal, pregnancy, preexisting diabetes, type 1 diabetes, type 2 diabetes

## Abstract

**Background/Objectives:** As the incidence of diabetes mellitus (DM) is increasing rapidly worldwide, it is anticipated that an increasing number of women will enter pregnancy with pregestational diabetes mellitus (PGDM) in the future. Compelling evidence suggests that hyperglycemia in pregnancy is related to multiple adverse perinatal outcomes. This systematic review and meta-analysis aims to assess and quantify the association of PGDM with a range of adverse perinatal outcomes, providing a comprehensive understanding of its impact on pregnancy. **Methods**: The data sources of this systematic review and meta-analysis were Medline/PubMed, Scopus and Cochrane Library (January 1999 to August 2023), complemented by hand-searching for additional references. Observational studies reporting perinatal outcomes of pregnancies with PGDM diagnosed before pregnancy versus control pregnancies were eligible for inclusion. A systematic review and meta-analysis were conducted as per the PRISMA guidelines. Pooled estimate odds ratios (ORs) with 95% confidence intervals (CIs) were calculated to determine the risk of adverse pregnancy outcomes between PGDM and control pregnancies. **Results**: The systematic search of the literature yielded 81 observational studies meeting inclusion criteria and in total, 137,237,640 pregnancies were included in the analysis. A total of 19 adverse perinatal outcomes were assessed, revealing a significant association with PGDM. In pregnancies with PGDM there was an increased risk of adverse perinatal outcomes, including gestational hypertension (OR 3.16, 95% CI 2.65–3.77), preeclampsia (OR 4.46, 95% CI 3.94–5.05), preterm delivery (OR 3.46, 95% CI 3.06–3.91), cesarean delivery (OR 3.12, 95% CI 2.81–3.47), induction of labor (OR 2.92, 95% CI 2.35–3.63), macrosomia (OR 2.23, 95% CI 1.76–2.83), LGA neonates (OR 3.95, 95% CI 3.47–4.49), low 5-min Apgar score (OR 2.49, 95% CI 2.07–2.99), shoulder dystocia (OR 3.05, 95% CI 2.07–4.50), birth trauma (OR 1.40, 95% CI 1.22–1.62), polyhydramnios (OR 5.06, 95% CI 4.33–5.91), oligohydramnios (OR 1.61, 95% CI 1.19–2.17), neonatal hyperbilirubinemia (OR 3.45, 95% CI 2.51–4.74), neonatal hypoglycemia (OR 19.19, 95% CI 2.78–132.61), neonatal intensive care unit (NICU) admission (OR 4.54, 95% CI 3.87–5.34), congenital malformations (OR 2.44, 95% CI 1.96–3.04), stillbirth (OR 2.87, 95% CI 2.27–3.63) and perinatal mortality (OR 2.94, 95% CI 2.18–3.98). Subgroup analyses indicated a higher risk of neonatal hypoglycemia, stillbirth and perinatal mortality in T1DM pregnancies compared with T2DM pregnancies. **Conclusions**: This study provides a robust synthesis of evidence underlying the strong association between PGDM and several adverse perinatal outcomes. Early detection, optimal glycemic control during the periconceptional and pregnancy periods, and proper antenatal care are critical to mitigate these risks.

## 1. Introduction

Diabetes mellitus (DM) seems to be one of the fastest-growing health issues of the 21st century; its global prevalence has more than doubled over the last four decades in the adult population, rising from 4.7% in 1980 to 10.5% in 2021 and it is estimated that the global prevalence will continue increasing, reaching 11.3% by 2030 [1,2]. DM is primarily classified into two types: type 1 diabetes mellitus (T1DM), which often develops in early life and is associated with a genetic predisposition, and type 2 diabetes mellitus (T2DM), which usually develops later in life and is more commonly associated with lifestyle factors. The reasons for the increase in T1DM prevalence are unclear but may involve a combination of environmental changes and altered early life factors (viral infections, gut microbiome) [3]. In contrast, the reasons for the rise in T2DM prevalence are more clearly understood, with increasingly sedentary lifestyles and rising obesity rates being the primary causes [4].

As a result of this trend and the increasing maternal age, more women will likely enter pregnancy with pregestational diabetes mellitus (PGDM) in the future. Compelling evidence demonstrates that hyperglycemia during pregnancy is related to an increased risk of adverse perinatal outcomes, such as preeclampsia, preterm delivery, macrosomia and congenital malformations [5,6,7,8,9,10]. Consequently, the number of pregnancies at risk is expected to rise alongside the increasing prevalence of DM.

Though many adverse perinatal outcomes associated with PGDM may have severe implications, the specific impact of PGDM on these outcomes has not been comprehensively explored in the existing literature. Most current knowledge is derived from single observational studies rather than systematic reviews and meta-analyses [11]. To date, only one systematic review has addressed this topic, but it did not assess crucial outcomes, such as congenital malformations [12]. In contrast, gestational diabetes mellitus (GDM) and its adverse perinatal outcomes have been studied systematically on various occasions [13,14,15].

Given the importance of accurate risk estimation for preconception counseling and the optimization of antenatal care, there is a critical need for robust, comprehensive data on the perinatal risks related to PGDM. To address this gap, the aim of this study is to systematically review the literature and conduct a meta-analysis assessing the association between PGDM and a wide range of adverse perinatal outcomes. By integrating data from an unprecedented number of pregnancies across diverse populations, this study provides highly precise effect estimates with enhanced statistical power. Importantly, it also differentiates between the effects of T1DM and T2DM, allowing for a more detailed understanding of their distinct impact on perinatal risks. This comprehensive approach enhances the generalizability and clinical applicability of the findings, thereby providing healthcare professionals and pregnant women with robust, globally applicable evidence to support evidence-based care and improve maternal and neonatal health.

## 2. Materials and Methods

The present systematic review and meta-analysis complied with a prespecified protocol registered to the PROSPERO database (International Prospective Register of Systematic Reviews) with registration number CRD42023459730 on 1 September 2023. Additionally, it adheres to the PRISMA guidelines, designed for transparent reporting of systematic reviews and meta-analyses [16].

### 2.1. Eligibility Criteria

The studies included in this systematic review and meta-analysis were selected based on specific eligibility criteria. We exclusively considered observational studies comparing adverse perinatal outcomes in two distinct groups: pregnancies with PGDM diagnosed before pregnancy (study group) and pregnancies without PGDM or GDM in the current pregnancy (control group). PGDM was defined as DM diagnosed prior to conception. Studies were only included if they clearly differentiated PGDM from gestational diabetes mellitus (GDM). The eligible study period was from 1999 onwards; this period was selected to maximize the homogeneity of results, as at this time, the World Health Organization (WHO) proposed a change in the diagnostic value of fasting glucose concentrations for the diagnosis of DM to 126 mg/dL, with the proposed diagnostic value still being used to this day [17]. Only studies published in English were considered for inclusion. We excluded studies with insufficient data for interpretation, those lacking an appropriate comparison group and those that did not adequately differentiate between PGDM and GDM.

### 2.2. Outcomes

The outcomes assessed were divided into maternal and fetal/neonatal. Maternal outcomes included gestational hypertension (defined as systolic blood pressure ≥ 140 mm Hg and/or diastolic blood pressure ≥ 90 mm Hg, presenting after 20 weeks of gestation for the first time without proteinuria or any end-organ dysfunction) [18], preeclampsia (defined as systolic blood pressure ≥ 140 mm Hg and/or diastolic blood pressure ≥ 90 mm Hg, accompanied by new-onset proteinuria or significant end-organ dysfunction after 20 weeks of gestation) [18], preterm delivery (defined as delivery before completing 37 weeks of gestation) [19], cesarean delivery and induction of labor. Fetal/neonatal outcomes included macrosomia (defined as birth weight > 4000 g) [20], large for gestational age (LGA) neonates (defined as birthweight > 90th percentile for gestational age) [20], small for gestational age (SGA) neonates (defined as birth weight < 10th percentile for gestational age) [21], low 5-min Apgar score (defined as 5-min Apgar score < 7) [22], shoulder dystocia, birth trauma (defined as any physical injury of the neonate during labor, e.g., clavicle fracture, brachial plexus injury), neonatal hyperbilirubinemia, neonatal hypoglycemia, admission to the neonatal intensive care unit (NICU), congenital malformations, stillbirth (defined as delivery of a fetus not exhibiting signs of life at or after 20 weeks of gestation) [23] and perinatal mortality.

### 2.3. Search Strategy and Information Sources

We aimed to identify observational studies assessing the effect of PGDM on adverse perinatal outcomes compared to control pregnancies without PGDM or GDM. The electronic databases searched were MEDLINE/PubMed, Scopus and Cochrane Library from January 1999 to August 2023. The last search was conducted on the 1st of September 2023. Each search used combinations of free-text and Medical Subject Heading (MeSH) terms combined with Boolean operators. The search syntax utilized in each database can be found in Appendix A. The results were supplemented with a manual search of reference lists of relevant publications and a grey literature search. Although we did not consult a professional librarian during the design of the search strategy, the search terms and strategies were carefully developed and pilot-tested by the authors (medical doctors with experience in systematic reviews) to ensure sensitivity and comprehensiveness.

### 2.4. Study Selection

All studies derived from the initial search were imported into Systematic Review Accelerator (https://sr-accelerator.com/ accessed on 1 September 2023), an online reference management tool provided by the Institute for Evidence-Based Healthcare of the University of Bond, and duplicates were removed. After deduplication, titles and abstracts of the studies were screened using the same tool by three independent reviewers (DG, AT, GK—medical doctors) to determine the eligibility of the studies against the eligibility criteria. Studies were considered eligible for full-text review and data extraction if they were observational studies with available full-text comparing perinatal outcomes in pregnancies with PGDM and control pregnancies. Full texts of potentially eligible studies were examined independently by two reviewers (DG, AT) to end up with the list of studies to be included in the systematic review and meta-analysis. If two or more studies used the same database for overlapping periods, only data from the study with the largest population were used. Disagreements between the reviewers were resolved by consensus.

### 2.5. Data Extraction

After the final selection of the eligible studies, data extraction forms were developed independently in Covidence (https://www.covidence.org/ accessed on 10 November 2023), an online tool for systematic review management, by two reviewers (DG, AT). Discrepancies were resolved by consensus. The extracted data included administrative characteristics of the studies, such as authors, year of publication and country where the study took place, design characteristics of the studies, such as type of study and sample size, baseline characteristics of the study populations, such as type of PGDM, as well as intervention characteristics, such as type of treatment during pregnancy. For each outcome of interest, the raw data (number of cases and the total population) were recorded for both the study and control groups.

### 2.6. Risk of Bias Assessment

The risk of bias for each study included in this systematic review and meta-analysis was assessed independently by two reviewers (DG, AT) using the Newcastle–Ottawa scale. The scale consists of three domains, each addressing distinct aspects of study quality using a “star system” for quality quantification [24]. The first domain assesses the selection of study groups, the second the comparability of these groups and the third domain appraises the ascertainment of either the outcome in cohort studies or the exposure in case-control studies. The studies included in this systematic review and meta-analysis were labeled as having a low risk of bias if they scored four stars for selection, two for comparison and three for outcome/exposure. Any study with a score of one or zero for the selection or outcome/exposure assessment or zero for the comparison assessment was considered to have a high risk of bias. In all other cases, the overall risk of bias was considered “unclear”. Disagreements between the reviewers were resolved by consensus.

### 2.7. Data Synthesis

The outcome data were dichotomous, so each group’s number of events and total participants were extracted for every outcome available. The odds ratio (OR) with 95% confidence intervals (CIs) was used as the effect measure to determine the likelihood of adverse pregnancy outcomes between PGDM and control pregnancies. Statistical heterogeneity was evaluated with Chi^2^ and quantified with the I^2^ statistics test. For outcomes with low heterogeneity (I^2^ ≤ 50%), the fixed effect model was used, and for outcomes with high heterogeneity (I^2^ > 50%), the random effect model was used. Potential sources of heterogeneity were explored with subgroup analyses investigating the effect of the different types of PGDM (T1DM and T2DM) on adverse perinatal outcomes. The Review Manager (RevMan) Version 5.4.1 was used for the statistical analysis of the results.

## 3. Results

### 3.1. Study Selection and Study Characteristics

**Figure 1:**
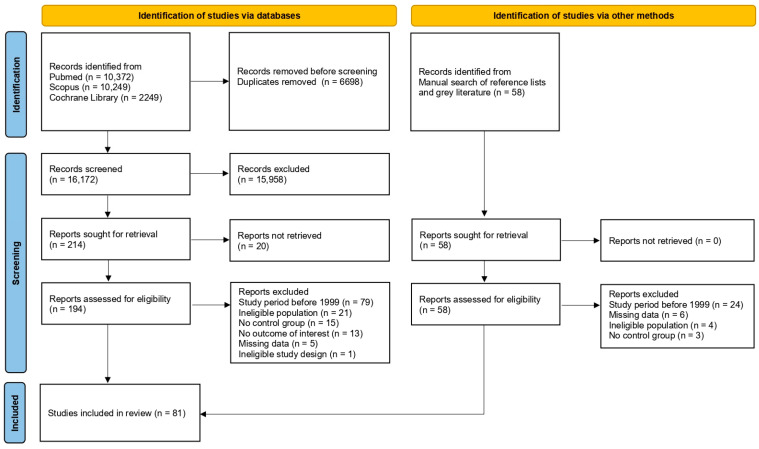
Study selection flowchart.

A total of 22,870 records were identified by the initial systematic search (10,372 via PubMed, 10,249 via Scopus and 2249 via Cochrane Library). After removing duplicate records, 16,172 were screened by title and abstract, and 214 were deemed suitable for full-paper appraisal; 194 full-text reports were retrieved, while the remaining 20 could not be obtained, even after a request was sent to the corresponding authors. Following the assessment of eligibility, 134 reports were excluded due to the following reasons: study period before 1999 (n = 79), ineligible population (n = 21), no control group (n = 15), no outcome of interest (n = 13), missing data (n = 5) and ineligible study design (n = 1). A manual search of reference lists of relevant publications and a search of grey literature identified 58 records suitable for full-paper appraisal. Full-text reports were retrieved for all of them. After the assessment of eligibility, 37 reports were excluded for the following reasons: study period before 1999 (n = 24), missing data (n = 6), ineligible population (n = 4) and no control group (n = 3). In total, 81 studies were included in the present meta-analysis [25,26,27,28,29,30,31,32,33,34,35,36,37,38,39,40,41,42,43,44,45,46,47,48,49,50,51,52,53,54,55,56,57,58,59,60,61,62,63,64,65,66,67,68,69,70,71,72,73,74,75,76,77,78,79,80,81,82,83,84,85,86,87,88,89,90,91,92,93,94,95,96,97,98,99,100,101,102,103,104,105]. A flow diagram illustrates the complete review process (Figure 1).

All 81 studies included were observational in design. Of these, 71 were cohort studies (60 were retrospective and 11 were prospective), while 10 were case-control studies. A total of 11 studies included only T1DM in their PGDM group, 8 included only T2DM and the remaining 62 included both T1DM and T2DM.

In total, 137,237,640 pregnancies were examined, including 1,151,826 pregnancies with PGDM and 136,085,814 control pregnancies. The studies were conducted at different times and locations, indicating no overlap in study populations. The studies were carried out in North America (26 studies), Europe (25 studies), East Asia (10 studies), the Middle East (8 studies), Oceania (10 studies) and South America (2 studies). A detailed table with the characteristics of the included studies can be found in Appendix A (Table A1).

### 3.2. Risk of Bias Assessment of the Included Studies

**Figure 2:**
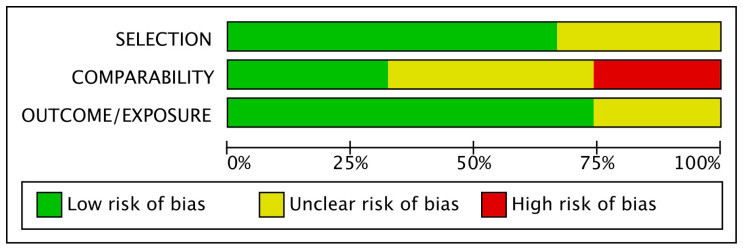
Risk of bias graph.

Based on the Newcastle–Ottawa scale, out of the 81 included studies, 13 were characterized as low risk, 47 as unclear risk and 21 as high risk. As shown in Figure 2, comparability was the most bias-prone domain among studies. Only a few studies matched their populations and adjusted their results for body mass index (BMI), which is considered the most important confounding factor for adverse perinatal outcomes. In contrast, other confounding factors, such as maternal age, were more frequently used for matches between groups and adjustments of results. At the same time, on many occasions, no confounder was considered.

### 3.3. PGDM and Adverse Perinatal Outcomes

The summary of adverse perinatal outcomes and the OR estimates for pregnancies with PGDM compared to control pregnancies are summarized in Table 1.

#### 3.3.1. Gestational Hypertension

**Figure 3:**
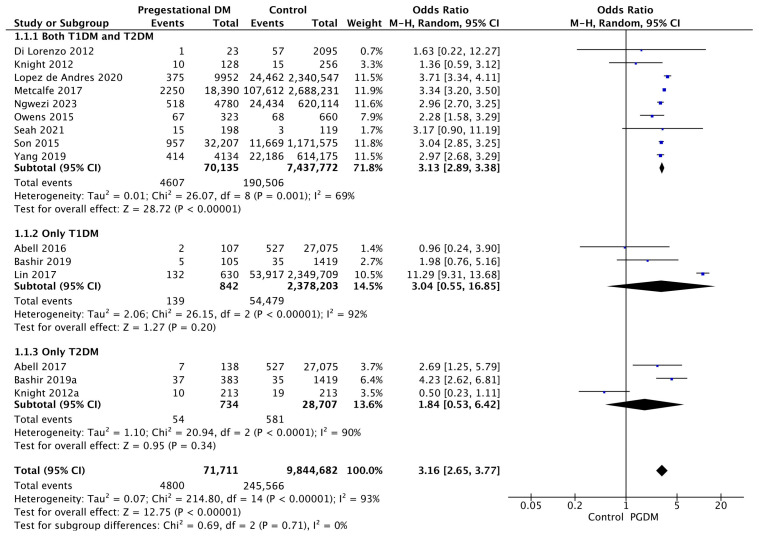
Forest plot comparing the incidence of gestational hypertension between PGDM and control groups [25,26,30,31,40,60,61,67,70,73,76,77,88,92,102].

Fifteen studies reported data on gestational hypertension, including 71,711 pregnant women with PGDM and 9,844,682 pregnant women without PGDM or GDM. In pregnancies with PGDM, the risk of gestational hypertension was increased compared to control pregnancies (OR 3.16, 95% CI 2.65–3.77, *p* < 0.00001, I^2^ = 93%). The subgroup analysis, including studies reporting data only for one type of PGDM, did not show a statistically significant difference in the risk of gestational hypertension between T1DM and T2DM (*p* = 0.64) (Figure 3).

#### 3.3.2. Preeclampsia

**Figure 4:**
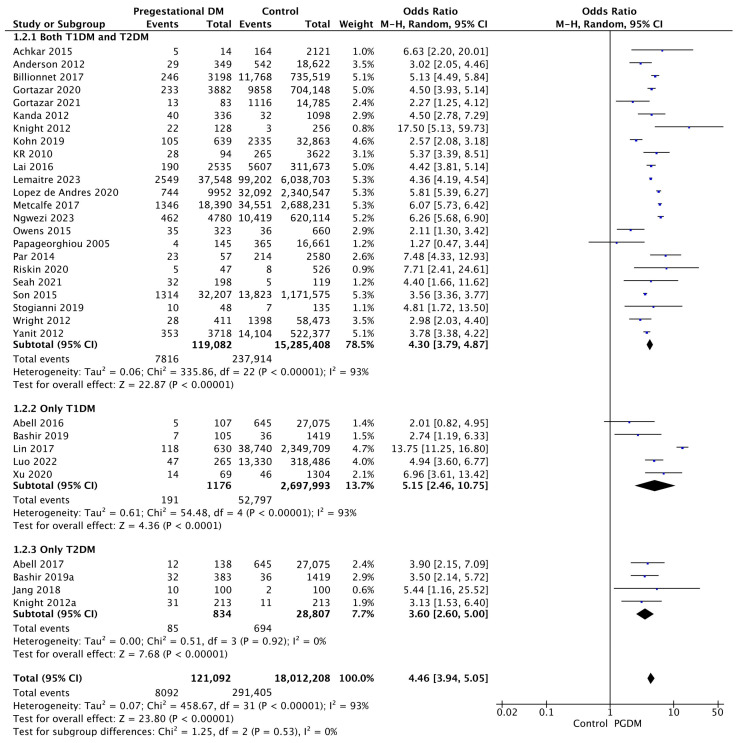
Forest plot comparing the incidence of preeclampsia between PGDM and control groups [25,26,27,28,30,31,35,47,50,51,55,57,60,61,62,64,66,67,70,72,73,76,77,78,79,86,88,92,94,99,101,103].

Thirty-two studies reported data on preeclampsia, including 121,092 pregnant women with PGDM and 18,012,208 pregnant women without PGDM or GDM. In pregnancies with PGDM, the risk of preeclampsia was increased compared to control pregnancies (OR 4.46, 95% CI 3.94–5.05, *p* < 0.00001, I^2^ = 93%). The subgroup analysis, including studies reporting data only for one type of PGDM, did not show a statistically significant difference in the risk of preeclampsia between T1DM and T2DM (*p* = 0.39) (Figure 4).

#### 3.3.3. Preterm Delivery

**Figure 5:**
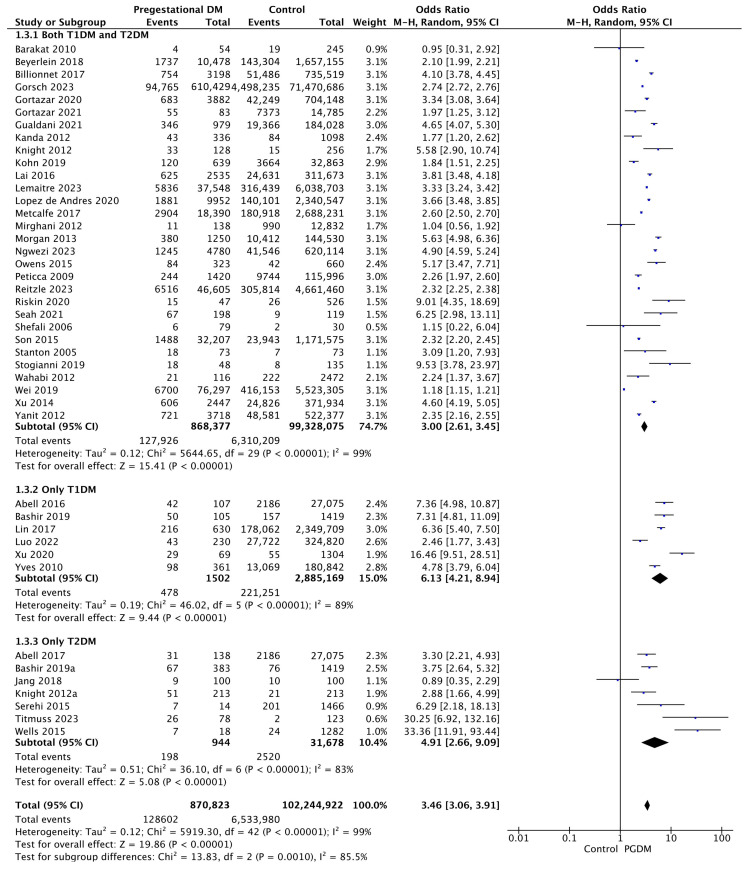
Forest plot comparing the incidence of preterm delivery between PGDM and control groups [25,26,29,30,31,33,35,49,50,51,52,55,57,60,61,62,64,66,67,70,72,73,74,75,76,77,82,85,86,88,89,90,92,93,94,95,96,97,98,100,101,103,104].

Forty-four studies reported data on preterm delivery, including 870,823 pregnant women with PGDM and 102,244,922 pregnant women without PGDM or GDM. In pregnancies with PGDM, the risk of preterm delivery was increased compared to control pregnancies (OR 3.46, 95% CI 3.06–3.91, *p* < 0.00001, I^2^ = 99%). The subgroup analysis, including studies reporting data only for one type of PGDM, did not show a statistically significant difference in the risk of preterm delivery between T1DM and T2DM (*p* = 0.55) (Figure 5).

#### 3.3.4. Cesarean Delivery

**Figure 6:**
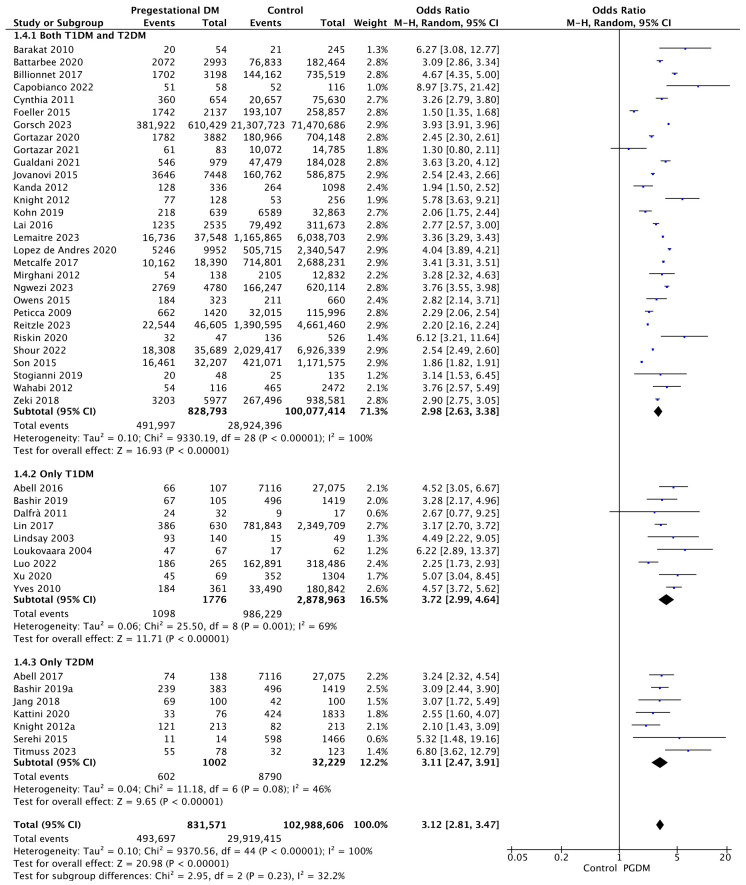
Forest plot comparing the incidence of cesarean delivery between PGDM and control groups [25,26,29,30,31,32,35,36,38,39,44,49,50,51,52,55,56,57,58,60,61,62,64,66,67,68,70,71,72,73,74,76,77,82,85,86,89,91,92,94,95,96,101,104,105].

Forty-five studies reported data on cesarean delivery, including 831,571 pregnant women with PGDM and 102,988,606 pregnant women without PGDM or GDM. In pregnancies with PGDM, the risk of cesarean delivery was increased compared to control pregnancies (OR 3.12, 95% CI 2.81–3.47, *p* < 0.00001, I^2^ = 100%). The subgroup analysis, including studies reporting data only for one type of PGDM, did not show a statistically significant difference in the risk of cesarean delivery between T1DM and T2DM (*p* = 0.27) (Figure 6).

#### 3.3.5. Induction of Labor

**Figure 7:**
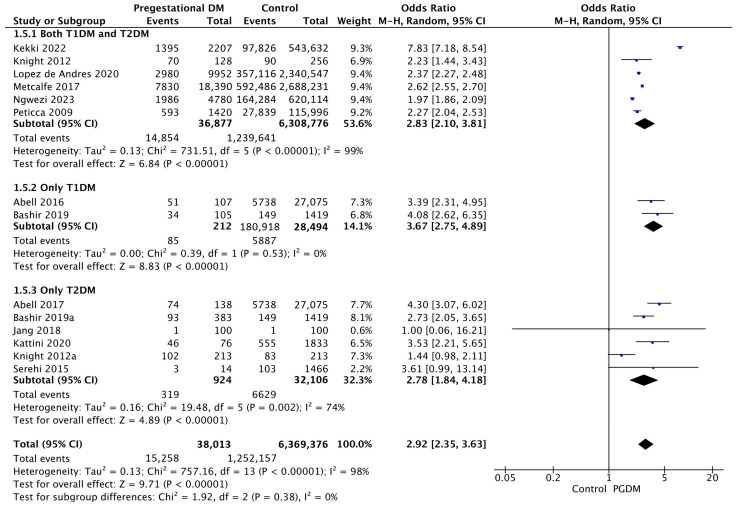
Forest plot comparing the incidence of induction of labor between PGDM and control groups [25,26,30,31,55,58,59,60,61,70,73,76,82,89].

Fourteen studies reported data on the induction of labor, including 38,013 pregnant women with PGDM and 6,369,376 pregnant women without PGDM or GDM. In pregnancies with PGDM, the risk of labor induction was increased compared to control pregnancies (OR 2.92, 95% CI 2.35–3.63, *p* < 0.00001, I^2^ = 98%). The subgroup analysis, including studies reporting data only for one type of PGDM, did not show a statistically significant difference in the risk of induction of labor between T1DM and T2DM (*p* = 0.28) (Figure 7).

#### 3.3.6. Macrosomia

**Figure 8:**
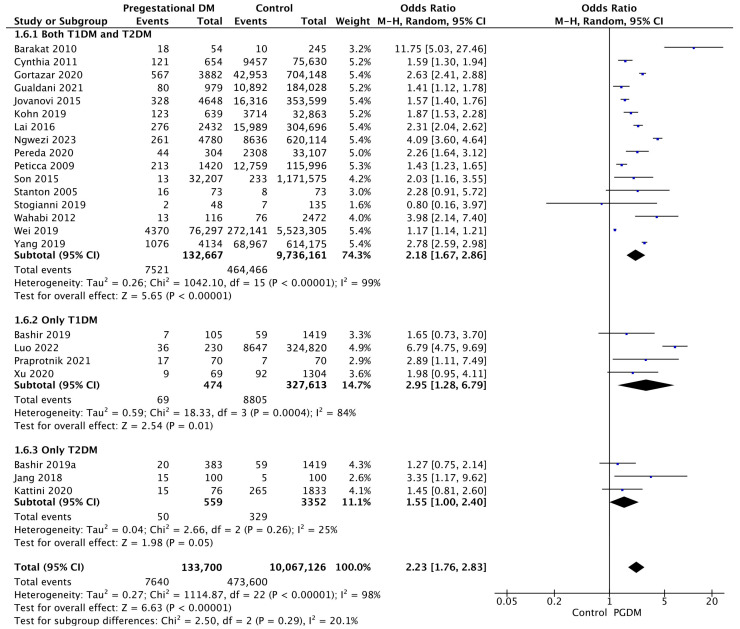
Forest plot comparing the incidence of macrosomia between PGDM and control groups [29,30,31,38,50,52,55,56,58,62,64,72,76,81,82,83,92,93,94,96,97,101,102].

Twenty-three studies reported data on macrosomia, including 133,700 pregnant women with PGDM and 10,067,126 pregnant women without PGDM or GDM. In pregnancies with PGDM, the risk of macrosomia was increased compared to control pregnancies (OR 2.23, 95% CI 1.76–2.83, *p* < 0.00001, I^2^ = 98%). The subgroup analysis, including studies reporting data only for one type of PGDM, did not show a statistically significant difference in the risk of macrosomia between T1DM and T2DM (*p* = 0.18) (Figure 8).

#### 3.3.7. LGA Neonates

**Figure 9:**
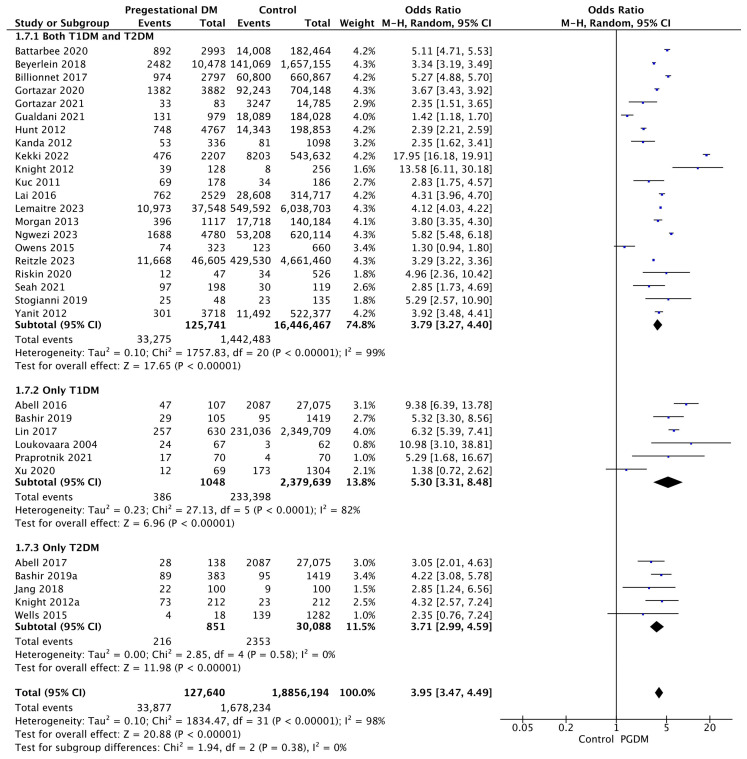
Forest plot comparing the incidence of LGA neonates between PGDM and control groups [25,26,30,31,32,33,35,50,51,52,54,55,57,59,60,61,63,64,66,67,71,75,76,77,83,85,86,88,94,98,101,103].

Thirty-two studies reported data on LGA neonates, including 127,640 pregnant women with PGDM and 18,856,194 pregnant women without PGDM or GDM. In pregnancies with PGDM, the risk of LGA neonates was increased compared to control pregnancies (OR 3.95, 95% CI 3.47–4.49, *p* < 0.00001, I^2^ = 98%). The subgroup analysis, including studies reporting data only for one type of PGDM, did not show a statistically significant difference in the risk of LGA neonates between T1DM and T2DM (*p* = 0.17) (Figure 9).

#### 3.3.8. SGA Neonates

**Figure 10:**
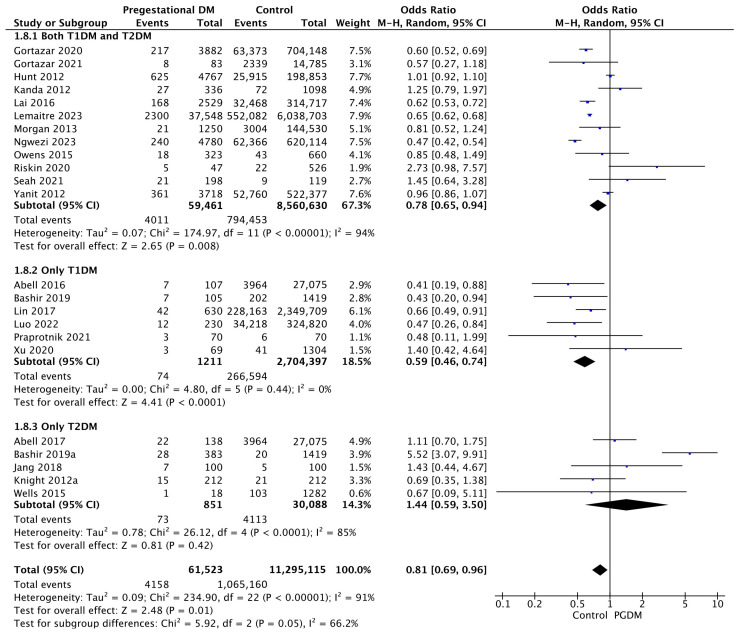
Forest plot comparing the incidence of SGA neonates between PGDM and control groups [25,26,30,31,50,51,54,55,57,61,64,66,67,72,75,76,77,83,86,88,98,101,103].

Twenty-three studies reported data on SGA neonates, including 61,523 pregnant women with PGDM and 11,295,115 pregnant women without PGDM or GDM. In pregnancies with PGDM, the risk of SGA neonates was decreased compared to control pregnancies (OR 0.81, 95% CI 0.69–0.96, *p* = 0.01, I^2^ = 91%). The subgroup analysis, including studies reporting data only for one type of PGDM, showed a statistically significant difference in the risk of SGA neonates between T1DM and T2DM, with a lower risk in the T1DM group (*p* = 0.06) (Figure 10).

#### 3.3.9. Low 5-Min Apgar Score

**Figure 11:**
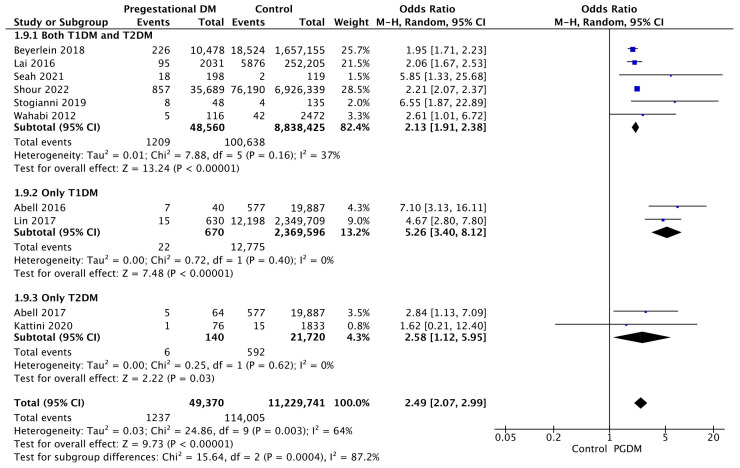
Forest plot comparing the incidence of low 5-min Apgar score between PGDM and control groups [25,26,33,58,64,67,88,91,94,96].

Ten studies reported data on low Apgar scores, including 49,370 pregnant women with PGDM and 11,229,741 pregnant women without PGDM or GDM. In pregnancies with PGDM, the risk of a low 5-min Apgar score was increased compared to control pregnancies (OR 2.49, 95% CI 2.07–2.99, *p* < 0.00001, I^2^ = 64%). The subgroup analysis, including studies reporting data only for one type of PGDM, did not show a statistically significant difference in the risk of low Apgar score between T1DM and T2DM (*p* = 0.14) (Figure 11).

#### 3.3.10. Shoulder Dystocia

**Figure 12:**
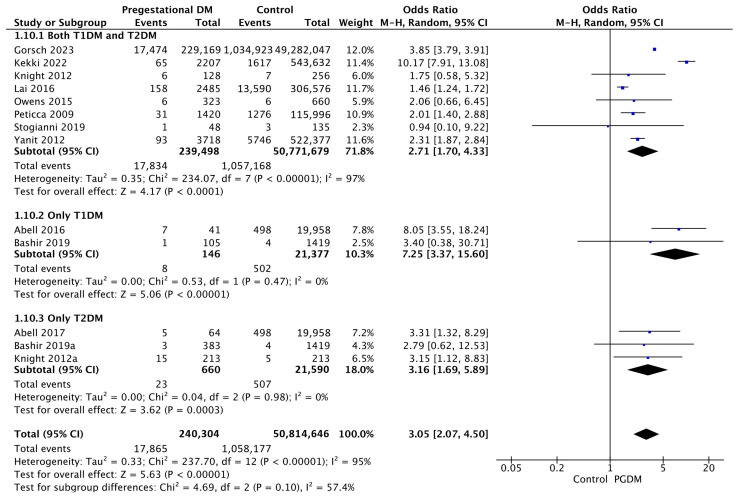
Forest plot comparing the incidence of shoulder dystocia between PGDM and control groups [25,26,30,31,49,59,60,61,64,77,82,94,103].

Thirteen studies reported data on shoulder dystocia, including 240,304 pregnant women with PGDM and 50,814,646 pregnant women without PGDM or GDM. In pregnancies with PGDM, the risk of shoulder dystocia was increased compared to control pregnancies (OR 3.05, 95% CI 2.07–4.50, *p* < 0.00001, I^2^ = 95%). The subgroup analysis, including studies reporting data only for one type of PGDM, did not show a statistically significant difference in the risk of shoulder dystocia between T1DM and T2DM (*p* = 0.10) (Figure 12).

#### 3.3.11. Birth Trauma

**Figure 13:**
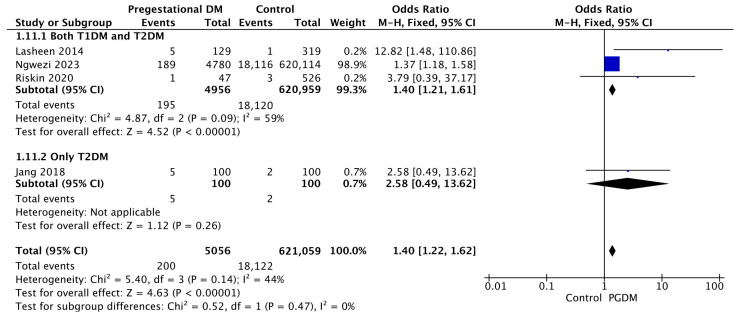
Forest plot comparing the incidence of birth trauma between PGDM and control groups [55,65,76,86].

Four studies reported data on birth trauma, including 5056 pregnant women with PGDM and 621,059 pregnant women without PGDM or GDM. In pregnancies with PGDM, the risk of birth trauma was increased compared to control pregnancies (OR 1.40, 95% CI 1.22–1.62, *p* < 0.00001, I^2^ = 44%). No studies reported data only for T1DM to allow a subgroup analysis between T1DM and T2DM (Figure 13).

#### 3.3.12. Polyhydramnios

**Figure 14:**
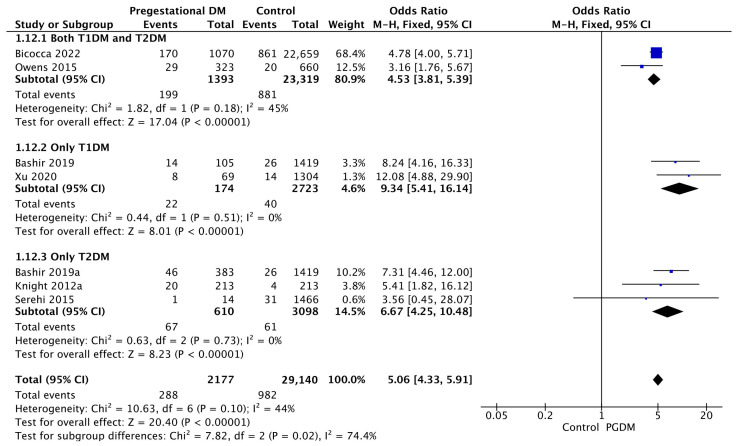
Forest plot comparing the incidence of polyhydramnios between PGDM and control groups [30,31,34,61,77,89,101].

Seven studies reported data on polyhydramnios, including 2177 pregnant women with PGDM and 29,140 pregnant women without PGDM or GDM. In pregnancies with PGDM, the risk of polyhydramnios was increased compared to control pregnancies (OR 5.06, 95% CI 4.33–5.91, *p* < 0.00001, I^2^ = 44%). The subgroup analysis, including studies reporting data only for one type of PGDM, did not show a statistically significant difference in the risk of polyhydramnios between T1DM and T2DM (*p* = 0.35) (Figure 14).

#### 3.3.13. Oligohydramnios

**Figure 15:**
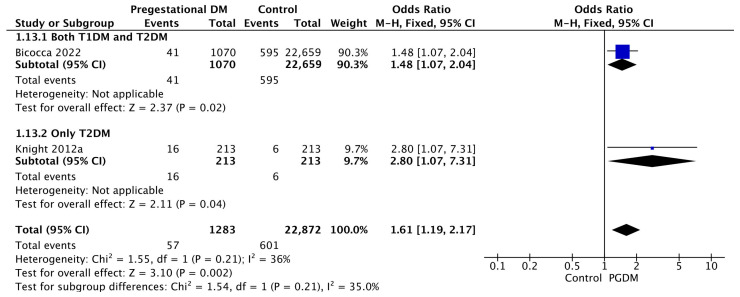
Forest plot comparing the incidence of oligohydramnios between PGDM and control groups [34,61].

Two studies reported data on oligohydramnios, including 1283 pregnant women with PGDM and 22,872 pregnant women without PGDM or GDM. In pregnancies with PGDM, the risk of oligohydramnios was increased compared to control pregnancies (OR 1.61, 95% CI 1.19–2.17, *p* = 0.002, I^2^ = 36%). No studies reported data only for T1DM to allow a subgroup analysis between T1DM and T2DM (Figure 15).

#### 3.3.14. Neonatal Hyperbilirubinemia

**Figure 16:**
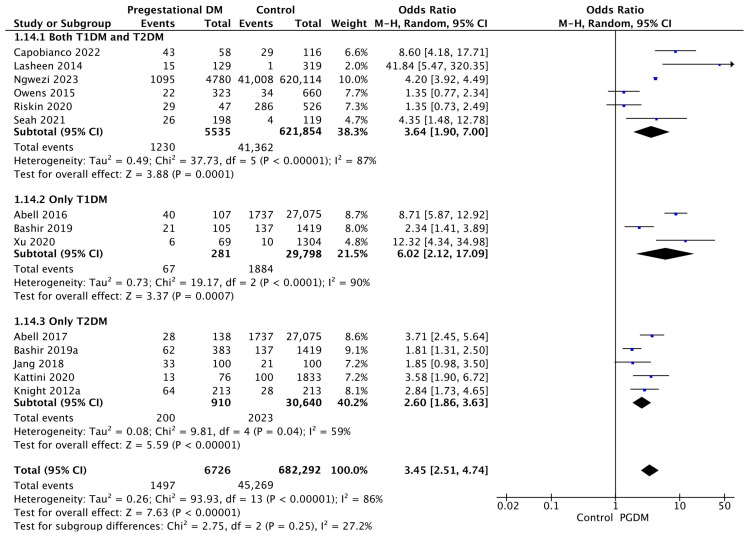
Forest plot comparing the incidence of neonatal hyperbilirubinemia between PGDM and control groups [25,26,30,31,36,55,58,61,65,76,77,86,88,101].

Fourteen studies reported data on neonatal hyperbilirubinemia, including 6726 pregnant women with PGDM and 682,292 pregnant women without PGDM or GDM. In pregnancies with PGDM, the risk of neonatal hyperbilirubinemia was increased compared to control pregnancies (OR 3.45, 95% CI 2.51–4.74, *p* < 0.00001, I^2^ = 86%). The subgroup analysis, including studies reporting data only for one type of PGDM, did not show a statistically significant difference in the risk of neonatal hyperbilirubinemia between T1DM and T2DM (*p* = 0.13) (Figure 16).

#### 3.3.15. Neonatal Hypoglycemia

**Figure 17:**
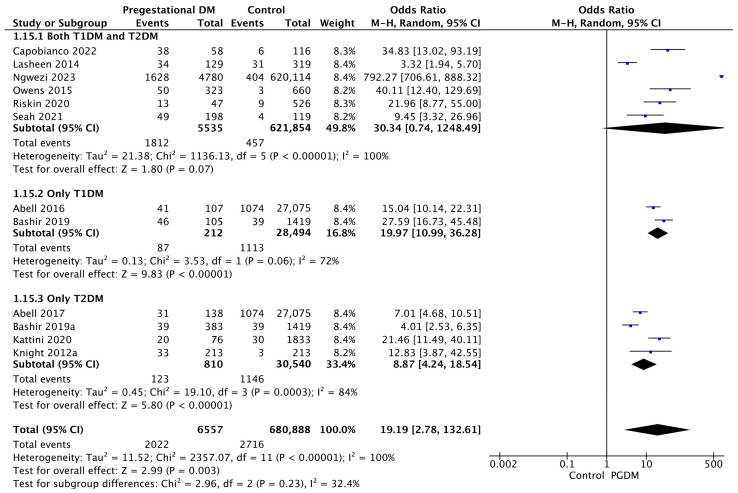
Forest plot comparing the incidence of neonatal hypoglycemia between PGDM and control groups [25,26,30,31,36,58,61,65,76,77,86,88].

Twelve studies reported data on neonatal hypoglycemia, including 6557 pregnant women with PGDM and 680,888 pregnant women without PGDM or GDM. In pregnancies with PGDM, the risk of neonatal hypoglycemia was increased compared to control pregnancies (OR 19.19, 95% CI 2.78–132.61, *p* = 0.003, I^2^ = 100%). However, the wide CI and high heterogeneity among the studies indicate significant variability, warranting cautious interpretation of these results. The subgroup analysis, including studies reporting data only for one type of PGDM, showed a statistically significant difference in the risk of neonatal hypoglycemia between T1DM and T2DM, with a higher risk in the T1DM group (*p* = 0.09) (Figure 17).

#### 3.3.16. NICU Admission

**Figure 18:**
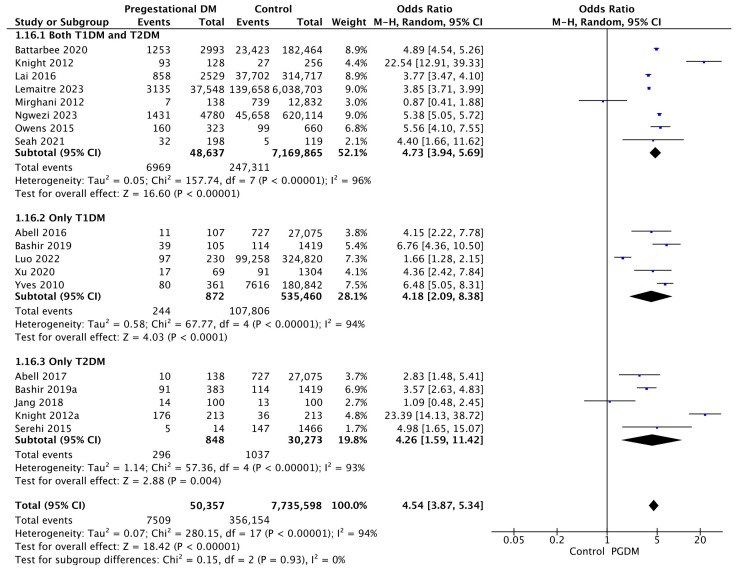
Forest plot comparing the incidence of NICU admission between PGDM and control groups [25,26,30,31,32,55,60,61,64,66,72,74,76,77,88,89,101,104].

Eighteen studies reported data on NICU admission, including 50,357 pregnant women with PGDM and 7,735,598 pregnant women without PGDM or GDM. In pregnancies with PGDM, the risk of NICU admission was increased compared to control pregnancies (OR 4.54, 95% CI 3.87–5.34, *p* < 0.00001, I^2^ = 94%). The subgroup analysis, including studies reporting data only for one type of PGDM, did not show a statistically significant difference in the risk of NICU admission between T1DM and T2DM (*p* = 0.98) (Figure 18).

#### 3.3.17. Congenital Malformations

**Figure 19:**
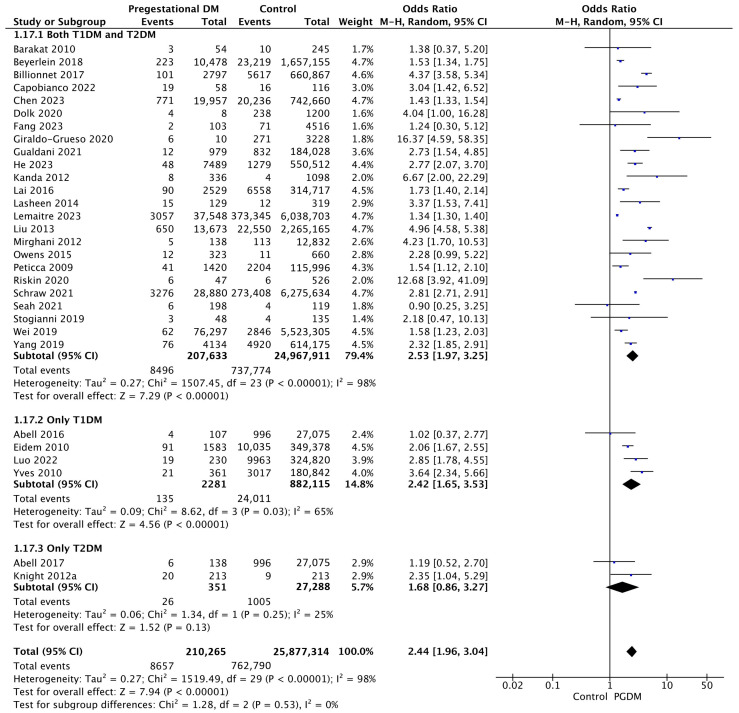
Forest plot comparing the incidence of congenital malformations between PGDM and control groups [25,26,29,33,35,36,37,41,42,43,46,52,53,57,61,64,65,66,69,72,74,77,82,86,87,88,94,97,102,104].

Thirty studies reported data on congenital malformations, including 210,265 pregnant women with PGDM and 25,877,314 pregnant women without PGDM or GDM. In pregnancies with PGDM, the risk of congenital malformation was increased compared to control pregnancies (OR 2.44, 95% CI 1.96–3.04, *p* < 0.00001, I^2^ = 98%). The subgroup analysis, including studies reporting data only for one type of PGDM, did not show a statistically significant difference in the risk of congenital malformation between T1DM and T2DM (*p* = 0.35) (Figure 19).

#### 3.3.18. Stillbirth

**Figure 20:**
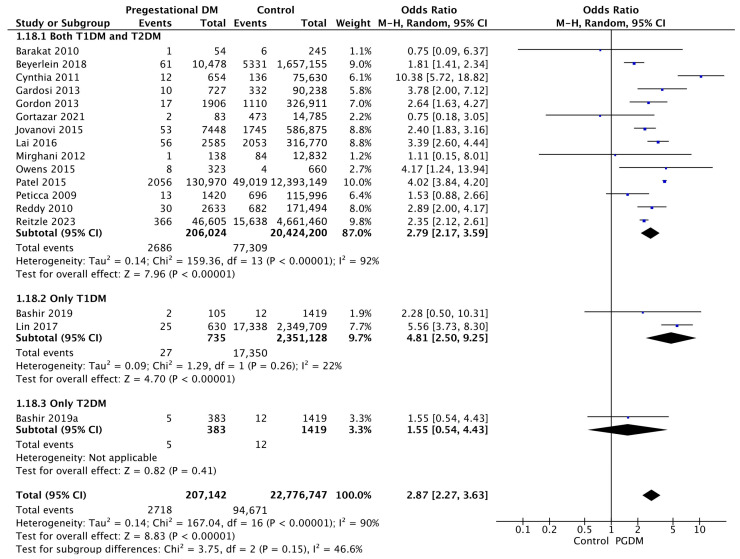
Forest plot comparing the incidence of stillbirth between PGDM and control groups [29,30,31,33,38,45,48,51,56,64,67,74,77,80,82,84,85].

Seventeen studies reported data on stillbirths, including 207,142 pregnant women with PGDM and 22,776,747 pregnant women without PGDM or GDM. In pregnancies with PGDM, the risk of stillbirth was increased compared to control pregnancies (OR 2.87, 95% CI 2.27–3.63, *p* < 0.00001, I^2^ = 90%). The subgroup analysis, including studies reporting data only for one type of PGDM, showed a statistically significant difference in the risk of stillbirth between T1DM and T2DM, with a higher risk in the T1DM group (*p* = 0.07) (Figure 20).

#### 3.3.19. Perinatal Mortality

**Figure 21:**
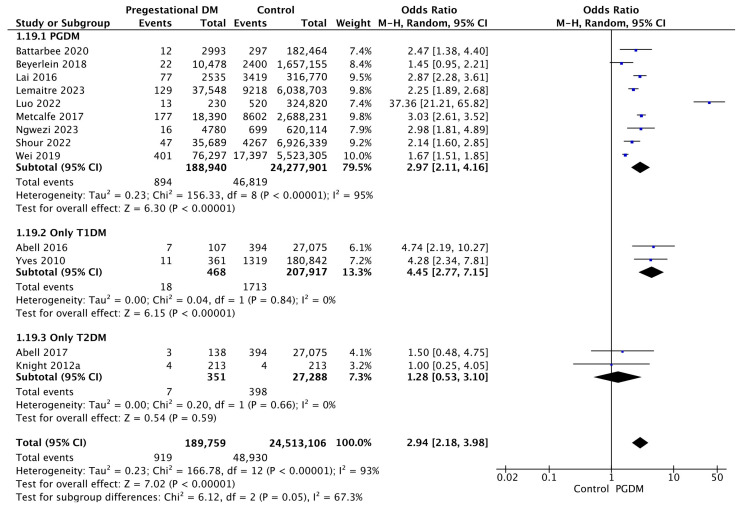
Forest plot comparing the incidence of perinatal mortality between PGDM and control groups [25,26,32,33,61,64,66,72,73,76,91,97,104].

Thirteen studies reported data on perinatal mortality, including 189,759 pregnant women with PGDM and 24,513,106 pregnant women without PGDM or GDM. In pregnancies with PGDM, the risk of perinatal mortality was increased compared to control pregnancies (OR 2.94, 95% CI 2.18–3.98, *p* < 0.00001, I^2^ = 93%). The subgroup analysis, including studies reporting data only for one type of PGDM, showed a statistically significant difference in the risk of perinatal mortality between T1DM and T2DM, with a higher risk in the T1DM group (*p* = 0.02) (Figure 21).

## 4. Discussion

### 4.1. Main Findings

The findings of the present systematic review and meta-analysis reveal that PGDM during pregnancy significantly increases the risk of various adverse perinatal outcomes compared to pregnancies without PGDM or GDM. The risk of each adverse outcome was quantified, allowing a comprehensive understanding of the impact of PGDM. More specifically, with regards to maternal adverse perinatal outcomes, the study demonstrated a significant positive correlation between PGDM and hypertensive disorders of pregnancy, including gestational hypertension and preeclampsia, as well as preterm delivery, cesarean delivery and induction of labor. Regarding fetal/neonatal outcomes, the study revealed that PGDM significantly increases the risk of macrosomia, LGA neonates, low 5-min Apgar score, shoulder dystocia, birth trauma, polyhydramnios, oligohydramnios, neonatal hyperbilirubinemia, neonatal hypoglycemia, NICU admission, congenital malformations, stillbirth and perinatal mortality. Notably, the risk of SGA neonates was found to be decreased in pregnancies with PGDM compared to pregnancies without PGDM or GDM. Subgroup analyses further showed that T1DM conferred a higher risk than T2DM for specific adverse outcomes, including neonatal hypoglycemia, stillbirth and perinatal mortality; for instance, perinatal mortality was quadrupled in T1DM pregnancies but did not increase significantly in those complicated by T2DM. Conversely, T1DM appeared to offer greater protection against SGA births in PGDM pregnancies compared to T2DM. For the remaining outcomes, based on the available data, no statistically significant differences emerged between T1DM and T2DM.

### 4.2. Comparison with Existing Literature

The findings of this study align with previous research, consistently demonstrating higher rates of adverse perinatal outcomes in pregnancies complicated by PGDM. Studies such as those by Abell et al. and Beyerlein et al. have similarly reported increased risks of adverse perinatal outcomes in pregnancies complicated by diabetes [25,33]. Moreover, studies by Schraw et al. and Lemaitre et al. highlighted the increased risk of congenital anomalies in neonates born to mothers with PGDM [66,87], while a study by Battarbee et al. found a particularly high risk of severe neonatal morbidity and mortality in pregnancies with PGDM [32]. The findings of our study are also in accord with reports by the International Diabetes Federation and the American Diabetes Association, both of which underscore the increased likelihood of poor pregnancy outcomes when PGDM is present [106,107].

To our knowledge, this study is the most comprehensive analysis to date examining the association between PGDM and adverse perinatal outcomes. Only one previous systematic review and meta-analysis by Yu et al., published in 2017, has addressed this topic [12]. It included 100 studies with data on around 40 million individuals and assessed 17 adverse outcomes. While its findings aligned with ours in reporting an increased risk of several adverse perinatal outcomes in pregnancies affected by PGDM, it did not identify an association between PGDM and SGA neonates. In contrast, our analysis demonstrated a decreased risk of SGA neonates in this group (OR 0.81, 95% CI 0.69–0.96). Furthermore, unlike our review, the earlier study did not assess several important outcomes evaluated in our study, such as congenital malformations (OR 2.44, 95% CI 1.96–3.04), induction of labor (OR 2.92, 95% CI 2.35–3.63), birth trauma (OR 1.40, 95% CI 1.22–1.62), polyhydramnios (OR 5.06, 95% CI 4.33–5.91) and oligohydramnios (OR 1.61, 95% CI 1.19–2.17). Additionally, the prior study did not conduct subgroup analyses by diabetes type, which in our review enabled direct comparisons between different forms of PGDM and their respective impacts on perinatal outcomes. Another key difference between the two studies is the sample size. Our study included a significantly larger sample size, incorporating data from over 137 million pregnancies, which enhances the statistical power and generalizability of our findings. We further strengthened our methodology by restricting inclusion to studies conducted from 1999 onwards, ensuring more uniform diagnostic criteria for PGDM and more consistent standards of pregnancy care across studies. These methodological choices position our review as the most current, robust and clinically relevant synthesis in the field.

### 4.3. Strengths and Limitations

This study has multiple strengths. It examines a broad spectrum of adverse perinatal outcomes across more than 137 million pregnancies, offering robust statistical power. Restricting the eligible study period to 1999 onwards ensured consistency in PGDM diagnostic criteria and allowed inclusion of more recent studies that reflect current care practices. Finally, by registering the protocol in a publicly accessible database before initiating the review, we promoted transparency and minimized bias in our methodology.

This study also has certain limitations. The estimation of risks of adverse perinatal outcomes based on pooled data for pregnancies with PGDM and control pregnancies is subject to the heterogeneity of the primary studies. The heterogeneity could be attributed to differences in study design, population demographics, methods of diagnosing PGDM and specialized pregnancy care for pregnant women with PGDM. The high level of heterogeneity noted for several adverse perinatal outcomes included in this study indicates significant methodological and clinical variation among the included studies, suggesting that the findings should be interpreted cautiously. Nevertheless, adopting a random effects model in the meta-analysis of the results may partially account for the within-study heterogeneity. Another limitation of this study is that there is no universal definition for some of the adverse perinatal outcomes studied (e.g., neonatal hypoglycemia). As a result, heterogeneous outcome definitions were used in the included studies. In addition, minor differences were observed in the offspring exclusion criteria among some studies; for instance, some excluded stillbirths or fetuses with chromosomal abnormalities from their populations, while others did not. Lastly, the restriction of eligibility to studies published only in English could be another limitation of this study. However, although there is a theoretical risk of excluding available evidence from this practice, empirical studies suggest that the impact of language bias on the findings of meta-analyses is likely negligible [108].

## 5. Conclusions

In conclusion, the present study contributes to a more comprehensive understanding of the strong correlation between PGDM and several adverse perinatal outcomes. The findings of this study underscore the necessity of early detection of PGDM in the preconception period and meticulous management of PGDM during pregnancy while facilitating evidence-based counseling for the affected population. It is well-established that achieving optimal glycemic control before pregnancy is crucial for mitigating the risks of PGDM during pregnancy. Future research should delve deeper into the pathophysiological mechanisms underlying PGDM-related complications and explore interventional strategies to determine the optimal timing and intensity of management. Moreover, large-scale, well-designed studies utilizing standardized outcome measures and controlling for potential confounding factors are required to quantify the risks of adverse perinatal outcomes more precisely.

## Figures and Tables

**Table 1:** Summary of findings.

Adverse Perinatal Outcome	Number of Studies	Odds Ratio [95% CI]	Pregnancies with PGDM	Pregnancies Without PGDM	*p*-Value	I^2^
Gestational hypertension	15	3.16 [2.65, 3.77]	71,711	9,844,682	*p* < 10^−5^	93%
Preeclampsia	32	4.46 [3.94, 5.05]	121,092	18,012,208	*p* < 10^−5^	93%
Preterm delivery	43	3.46 [3.06, 3.91]	870,823	102,244,922	*p* < 10^−5^	99%
Cesarean delivery	45	3.12 [2.81, 3.47]	831,571	102,988,606	*p* < 10^−5^	100%
Induction of labor	14	2.92 [2.35, 3.63]	38,013	6,369,376	*p* < 10^−5^	98%
Macrosomia	23	2.23 [1.76, 2.83]	133,700	10,067,126	*p* < 10^−5^	98%
LGA neonates	32	3.95 [3.47, 4.49]	127,640	18,856,194	*p* < 10^−5^	98%
SGA neonates	23	0.81 [0.69, 0.96]	61,523	11,295,115	*p* = 0.01	91%
Low 5-min Apgar score	10	2.49 [2.07, 2.99]	49,370	11,229,741	*p* < 10^−5^	64%
Shoulder dystocia	13	3.05 [2.07, 4.50]	240,304	50,814,646	*p* < 10^−5^	95%
Birth trauma	4	1.40 [1.22, 1.62]	5056	621,059	*p* < 10^−5^	44%
Polyhydramnios	7	5.06 [4.33, 5.91]	2177	29,140	*p* < 10^−5^	44%
Oligohydramnios	2	1.61 [1.19, 2.17]	1283	22,872	*p* = 0.002	36%
Neonatal hyperbilirubinemia	14	3.45 [2.51, 4.74]	6726	682,292	*p* < 10^−5^	86%
Neonatal hypoglycemia	12	19.19 [2.78, 132.61]	6557	680,888	*p* = 0.003	100%
NICU admission	18	4.54 [3.87, 5.34]	50,357	7,735,598	*p* < 10^−5^	94%
Congenital malformation	30	2.44 [1.96, 3.04]	210,265	25,877,314	*p* < 10^−5^	98%
Stillbirth	17	2.87 [2.27, 3.63]	207,142	22,776,747	*p* < 10^−5^	90%
Perinatal mortality	13	2.94 [2.18, 3.98]	189,759	24,513,106	*p* < 10^−5^	93%

Abbreviations: LGA = large for gestational age, NICU = neonatal intensive care unit, PGDM = pregestational diabetes mellitus, SGA = small for gestational age.

## Data Availability

The original contributions presented in this study are included in the article/Supplementary Material. Further inquiries can be directed to the corresponding author.

## Supplementary Materials

The following supporting information can be downloaded at https://www.mdpi.com/article/10.3390/jcm14134789/s1.

## Appendix A

**Table A1:** Characteristics of included studies.

Study	Country	Study Design	Types of PGDM Included	PGDM Pregnancies/Controls	Adverse Perinatal Outcomes Studied	Risk of Bias Score (NOS)
**Abell 2016 [25]**	Australia	Retrospective cohort study	T1DM	107/27,075	GH, PE, PD, CD, IoL, LGA, SGA, Low Apgar score, SD, NHB, NHG, NICU admission, CM, Perinatal mortality	9
**Abell 2017 [26]**	Australia	Retrospective cohort study	T2DM	138/27,075	GH, PE, PD, CD, IoL, LGA, SGA, Low Apgar score, SD, NHB, NHG, NICU admission, CM, Perinatal mortality	9
**Achkar 2015 [27]**	Canada	Case-control study	Not specified	14/2121	PE	6
**Anderson 2012 [28]**	New Zealand	Retrospective cohort study	T1DM, T2DM	349/18,622	PE	8
**Barakat 2010 [29]**	Oman	Retrospective cohort study	T1DM, T2DM	54/245	PD, CD, Macrosomia, CM, Stillbirth	8
**Bashir 2019 [30]**	Qatar	Retrospective cohort study	T2DM	383/1419	GH, PE, PD, CD, IoL, Macrosomia, LGA, SGA, SD, PH, NHB, NHG, NICU admission, Stillbirth	7
**Bashir 2019 [31]**	Qatar	Retrospective cohort study	T1DM	105/1419	GH, PE, PD, CD, IoL, Macrosomia, LGA, SGA, SD, PH, NHB, NHG, NICU admission, Stillbirth	7
**Battarbee 2020 [32]**	USA	Retrospective cohort study	T1DM, T2DM	2993/182,464	CD, LGA, NICU admission, Perinatal mortality	9
**Beyerlein 2018 [33]**	Germany	Retrospective cohort study	Not specified	10,478/1,657,155	PD, LGA, Low Apgar score, CM, Stillbirth, Perinatal mortality	9
**Bicocca 2022 [34]**	USA	Retrospective cohort study	T1DM, T2DM	1070/22,659	PH, OH	7
**Billionnet 2017 [35]**	France	Retrospective cohort study	T1DM, T2DM	3198/735,519	PE, PD, CD, LGA, CM	8
**Capobianco 2022 [36]**	Italy	Case-control study	T1DM, T2DM, MODY	58/116	CD, NHB, NHG, CM	7
**Chen 2023 [37]**	Taiwan	Retrospective cohort study	T1DM, T2DM	19,957/742,660	CM	8
**Cynthia 2011 [38]**	Australia	Retrospective cohort study	Not specified	654/75,630	CD, Macrosomia, Stillbirth	6
**Dalfrà 2011 [39]**	Italy	Retrospective cohort study	T1DM	32/17	CD	4
**Di Lorenzo 2012 [40]**	Italy	Prospective cohort study	Not specified	23/2095	GH	5
**Dolk 2020 [41]**	UK	Case-control study	Not specified	8/1200	CM	7
**Eidem 2010 [42]**	Norway	Retrospective cohort study	T1DM	1583/349,378	CM	8
**Fang 2023 [43]**	Taiwan	Case-control study	Not specified	103/4516	CM	7
**Foeller 2015 [44]**	USA	Retrospective cohort study	T1DM, T2DM	2137/258,857	CD	8
**Gardosi 2013 [45]**	UK	Prospective cohort study	Not specified	727/90,238	Stillbirth	9
**Giraldo-Grueso 2020 [46]**	Colombia	Case-control study	Not specified	10/3228	CM	5
**Goetzinger 2010 [47]**	USA	Retrospective cohort study	Not specified	94/3622	PE	6
**Gordon 2013 [48]**	Australia	Prospective cohort study	Not specified	1906/326,911	Stillbirth	8
**Gorsch 2023 [49]**	USA	Retrospective cohort study	T1DM, T2DM	610,429/71,470,686	PD, CD, SD	8
**Gortazar 2020 [50]**	Spain	Retrospective cohort study	T1DM, T2DM, other PGDM types	3882/704,148	PE, PD, CD, Macrosomia, LGA, SGA	8
**Gortazar 2021 [51]**	Spain	Retrospective cohort study	T1DM, T2DM, other PGDM types	83/14,785	PE, PD, CD, LGA, SGA, Stillbirth	8
**Gualdani 2021 [52]**	Italy	Retrospective cohort study	T1DM, T2DM	979/184,028	PD, CD, Macrosomia, LGA, CM	9
**He 2023 [53]**	Canada	Retrospective cohort study	Not specified	7489/550,512	CM	9
**Hunt 2012 [54]**	USA	Retrospective cohort study	Not specified	4767/198,853	LGA, SGA	9
**Jang 2018 [55]**	Korea	Case-control study	T2DM	100/100	PE, PD, CD, IoL, Macrosomia, LGA, SGA, BT, NHB, NICU admission	6
**Jovanovič 2015 [56]**	USA	Retrospective cohort study	T1DM, T2DM	11,261/773,751	CD, Macrosomia, Stillbirth	7
**Kanda 2012 [57]**	Japan	Retrospective cohort study	T1DM, T2DM	336/1098	PE, PD, CD, LGA, SGA, CM	8
**Kattini 2020 [58]**	Canada	Retrospective cohort study	T2DM	76/1833	CD, IoL, Macrosomia, Low Apgar score, NHB, NHG	7
**Kekki 2022 [59]**	Finland	Retrospective cohort study	T1DM, T2DM	2207/543,632	IoL, LGA, SD	8
**Knight 2012 [60]**	USA	Retrospective cohort study	T1DM, T2DM	128/256	GH, PE, PD, CD, IoL, LGA, SD, NICU admission	5
**Knight 2012 [61]**	USA	Retrospective cohort study	T2DM	213/213	GH, PE, PD, CD, IoL, LGA, SGA, SD, PH, OH, NHB, NHG, NICU admission, CM, Perinatal mortality	9
**Kohn 2019 [62]**	USA	Retrospective cohort study	Not specified	639/32,863	PE, PD, CD, Macrosomia	8
**Kuc 2011 [63]**	Netherlands	Case-control study	Not specified	178/186	LGA	6
**Lai 2016 [64]**	Canada	Retrospective cohort study	T1DM, T2DM	2535/311,673	PE, PD, CD, Macrosomia, LGA, SGA, Low Apgar score, SD, NICU admission, CM, Stillbirth, Perinatal mortality	8
**Lasheen 2014 [65]**	Saudi Arabia	Prospective cohort study	Not specified	129/319	BT, NHB, NHG, CM	6
**Lemaitre 2023 [66]**	France	Retrospective cohort study	T1DM, T2DM	37,548/6,038,703	PE, PD, CD, LGA, SGA, NICU admission, CM, Perinatal mortality	8
**Lin 2017 [67]**	Taiwan	Retrospective cohort study	T1DM	630/2,349,709	GH, PE, PD, CD, LGA, SGA, Low Apgar score, Stillbirth	8
**Lindsay 2003 [68]**	UK	Case-control study	T1DM	140/49	CD	6
**Liu 2013 [69]**	Canada	Retrospective cohort study	T1DM, T2DM	13,673/2,265,165	CM	8
**Lopez-de-Andres 2020 [70]**	Spain	Retrospective cohort study	T1DM, T2DM	9952/2,340,547	GH, PE, PD, CD, IoL	8
**Loukovaara 2004 [71]**	Finland	Retrospective cohort study	T1DM	67/62	CD, LGA	6
**Luo 2022 [72]**	China	Retrospective cohort study	T1DM	265/318,486	PE, PD, CD, Macrosomia, SGA, NICU admission, CM, Perinatal mortality	7
**Metcalfe 2017 [73]**	Canada	Retrospective cohort study	T1DM, T2DM	18,390/2,688,231	GH, PE, PD, CD, IoL, Perinatal mortality	8
**Mirghani 2012 [74]**	UAE	Prospective cohort study	T1DM, T2DM	138/12,832	PD, CD, NICU admission, CM, Stillbirth	5
**Morgan 2013 [75]**	UK	Retrospective cohort study	T1DM, T2DM	1250/144,530	PD, LGA, SGA	8
**Ngwezi 2023 [76]**	Canada	Retrospective cohort study	T1DM, T2DM	4780/620,114	GH, PE, PD, CD, IoL, Macrosomia, LGA, SGA, BT, NHB, NHG, NICU admission, Perinatal mortality	8
**Owens 2015 [77]**	Ireland	Case-control study	T1DM, T2DM	323/660	GH, PE, PD, CD, LGA, SGA, SD, PH, NHB, NHG, NICU admission, CM, Stillbirth	6
**Papageorghiou 2005 [78]**	UK	Prospective cohort study	Not specified	145/16,661	PE	6
**Paré 2014 [79]**	USA	Prospective cohort study	Not specified	57/2580	PE	8
**Patel 2015 [80]**	USA	Case-control study	Not specified	130,970/12,393,149	Stillbirth	8
**Pereda 2020 [81]**	Uruguay	Retrospective cohort study	Not specified	304/33,107	Macrosomia	9
**Peticca 2009 [82]**	Canada	Retrospective cohort study	T1DM, T2DM	1420/115,996	PD, CD, IoL, Macrosomia, SD, CM, Stillbirth	8
**Praprotnik 2021 [83]**	Croatia	Retrospective cohort study	T1DM	70/70	Macrosomia, LGA, SGA	5
**Reddy 2010 [84]**	USA	Retrospective cohort study	Not specified	2633/172,176	Stillbirth	8
**Reitzle 2023 [85]**	Germany	Retrospective cohort study	Not specified	46,605/4,661,460	PD, CD, LGA, Stillbirth	7
**Riskin 2020 [86]**	Israel	Case-control study	Not specified	47/526	PE, PD, CD, LGA, SGA, BT, NHB, NHG, CM	6
**Schraw 2021 [87]**	USA	Retrospective cohort study	Not specified	28,880/6,275,634	CM	9
**Seah 2021 [88]**	Australia	Retrospective cohort study	T1DM, T2DM	198/119	GH, PE, PD, LGA, SGA, Low Apgar score, NHB, NHG, NICU admission, CM	7
**Serehi 2015 [89]**	Saudi Arabia	Prospective cohort study	T2DM	14/1466	PD, CD, IoL, PH, NICU admission	7
**Shefali 2006 [90]**	India	Prospective cohort study	T1DM, T2DM	79/30	PD	7
**Shour 2022 [91]**	USA	Retrospective cohort study	Not specified	35,689/6,926,339	CD, Low Apgar score, Perinatal mortality	9
**Son 2015 [92]**	Korea	Retrospective cohort study	Not specified	32,207/1,171,575	GH, PE, PD, CD, Macrosomia	8
**Stanton 2005 [93]**	USA	Retrospective cohort study	Not specified	73/73	PD, Macrosomia	5
**Stogianni 2019 [94]**	Sweden	Retrospective cohort study	T1DM, T2DM	48/135	PE, PD, CD, Macrosomia, LGA, Low Apgar score, SD, CM	8
**Titmuss 2023 [95]**	Australia	Prospective cohort study	T2DM	78/123	PD, CD	8
**Wahabi 2012 [96]**	Saudi Arabia	Retrospective cohort study	T1DM, T2DM	116/2472	PD, CD, Macrosomia, Low Apgar score	8
**Wei 2019 [97]**	China	Retrospective cohort study	Not specified	76,297/5,523,305	PD, Macrosomia, CM, Perinatal mortality	8
**Wells 2015 [98]**	Australia	Retrospective cohort study	T2DM	18/1282	PD, LGA, SGA	6
**Wright 2012 [99]**	UK	Prospective cohort study	T1DM, T2DM	411/58,473	PE	8
**Xu 2014 [100]**	Australia	Retrospective cohort study	Not specified	2447/372,954	PD	8
**Xu 2020 [101]**	China	Retrospective cohort study	T1DM	69/1304	PE, PD, CD, Macrosomia, LGA, SGA, PH, NHB, NICU admission	8
**Yang 2019 [102]**	USA	Retrospective cohort study	Not specified	4134/614,175	GH, Macrosomia, CM	9
**Yanit 2012 [103]**	USA	Retrospective cohort study	Not specified	3718/522,377	PE, PD, LGA, SGA, SD	8
**Yves 2010 [104]**	Belgium	Retrospective cohort study	T1DM	354/177,407	PD, CD, NICU admission, CM, Perinatal mortality	7
**Zeki 2018 [105]**	Australia	Retrospective cohort study	T1DM, T2DM	5977/938,581	CD	8

Abbreviations: T1DM = type 1 diabetes mellitus, T2DM = type 2 diabetes mellitus, GH = gestational hypertension, PE = preeclampsia, PD = preterm delivery, CD = cesarean delivery, IoL = induction of labor, LGA = large for gestational age, SGA = small for gestational age, SD = shoulder dystocia, BT = birth trauma, PH = polyhydramnios, OH = oligohydramnios, NHB = neonatal hyperbilirubinemia, NHG = neonatal hypoglycemia, NICU = neonatal intensive care unit, CM = congenital malformation.

### Appendix A.1. MEDLINE/PubMed Search Strategy

MEDLINE/Pubmed search syntax (Advanced search)

#1: “pregnancy” [All Fields]
#2: “pregnant” [All Fields]
#3: #1 OR #2
#4: “diabetes” [All Fields]
#5: #3 AND #4
#6: “pregestational” [All Fields]
#7: “pre-gestational” [All Fields]
#8: “preexisting” [All Fields]
#9: “pre-existing” [All Fields]
#10: “type 1 diabetes” [All Fields]
#11: “diabetes type 1” [All Fields]
#12: type 1 diabetes mellitus [MesH Terms]
#13: “type 2 diabetes” [All Fields]
#14: “diabetes type 2” [All Fields]
#15: type 2 diabetes mellitus [MesH Terms]
#16: #6 OR #7 OR #8 OR #9 OR #10 OR #11 OR #12 OR #13 OR #14 OR #15
#17: #5 AND #16
Publication date: 1999 onwards

MEDLINE/PubMed search string

(((“pregnancy”) OR (“pregnant”)) AND (“diabetes”)) AND ((((((((((“pregestational”) OR (“pre-gestational”)) OR (“preexisting”)) OR (“pre-existing”)) OR (“type 1 diabetes”)) OR (“diabetes type 1”)) OR (type 1 diabetes mellitus [MeSH Terms])) OR (“type 2 diabetes”)) OR (“diabetes type 2”)) OR (type 2 diabetes mellitus [MeSH Terms]))
Publication date: 1999 onwards

### Appendix A.2. Scopus Search Strategy

Scopus search syntax (Advanced search)

#1: “pregnancy”
#2: “pregnant”
#3: #1 OR #2
#4: “diabetes” [All Fields]
#5: #3 AND #4
#6: “pregestational”
#7: “pre-gestational”
#8: “preexisting”
#9: “pre-existing”
#10: “type 1 diabetes”
#11: “diabetes type 1”
#12: “diabetes mellitus, type 1”
#13: “type 2 diabetes”
#14: “diabetes type 2”
#15: “diabetes mellitus, type 2”
#16: #6 OR #7 OR #8 OR #9 OR #10 OR #11 OR #12 OR #13 OR #14 OR #15
#17: #5 AND #16
Limited to Subject Area: Medicine
Limited to English
Publication date: 1999 onwards

Scopus search string

TITLE-ABS-KEY ((“pregnancy” OR “pregnant”) AND “diabetes” AND (“pregestational” OR “pre-gestational” OR “preexisting” OR “pre-existing” OR “type 1 diabetes” OR “diabetes type 1” OR “diabetes mellitus, type 1” OR “type 2 diabetes” OR “diabetes type 2” OR “diabetes mellitus, type 2”)) AND PUBYEAR > 1998 AND PUBYEAR < 2024 AND (LIMIT-TO (SUBJAREA, “MEDI”)) AND (LIMIT-TO (LANGUAGE, “English”))

### Appendix A.3. Cochrane Library Search Strategy

Cochrane Library search syntax

#1: “pregnancy”
#2: “pregnant”
#3: #1 OR #2
#4: “diabetes”
#5: #3 AND #4
#6: “pregestational”
#7: “pre-gestational”
#8: “preexisting”
#9: “pre-existing”
#10: “type 1 diabetes”
#11: “diabetes type 1”
#12: MeSH descriptor: [Diabetes Mellitus, Type 1] explode all trees
#13: “type 2 diabetes”
#14: “diabetes type 2”
#15: MeSH descriptor: [Diabetes Mellitus, Type 2] explode all trees
#16: #6 OR #7 OR #8 OR #9 OR #10 OR #11 OR #12 OR #13 OR #14 OR #15
#17: #5 AND #16
Publication date: 1999 onwards
