# Supplementary material for: Pregestational Diabetes Mellitus and Adverse Perinatal Outcomes: A Systematic Review and Meta-Analysis
Source: J Clin Med. 2025 Jul 7;14(13):4789. doi: 10.3390/jcm14134789 (PMC12251443; doi:10.3390/jcm14134789)
Supplement: Supplementary file 1 [file jcm-14-04789-s001.zip › PGDM_supplementary/PGDM_Funnel_plots.pdf]

Funnel plots of comparison: PGDM vs Control

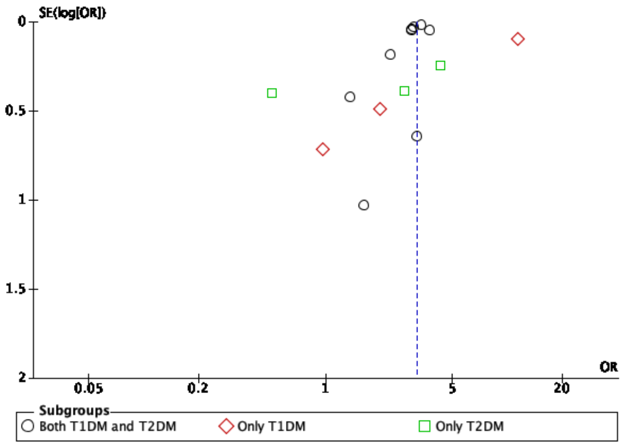

Supplementary Figure S1. Gestational hypertension

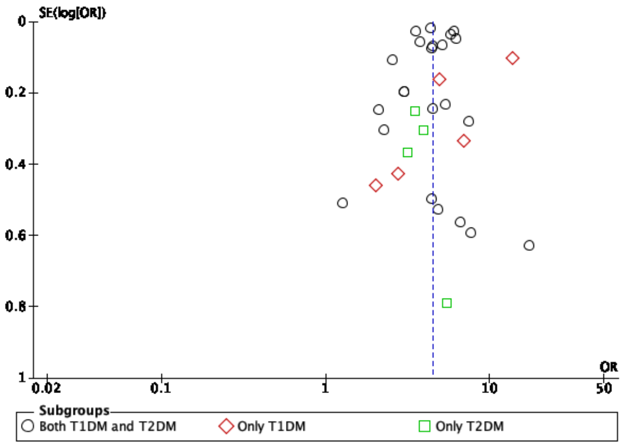

Supplementary Figure S2. Preeclampsia

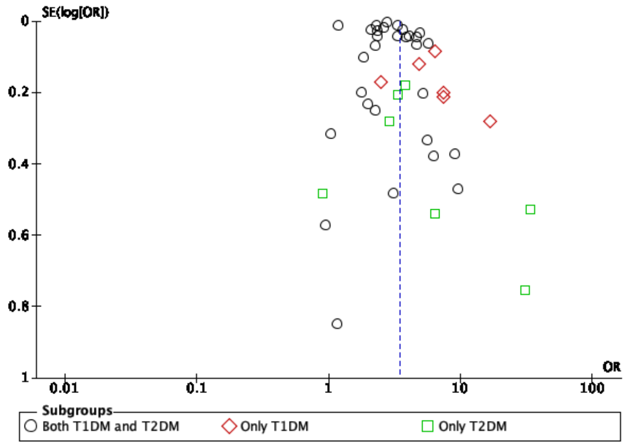

Supplementary Figure S3. Preterm delivery

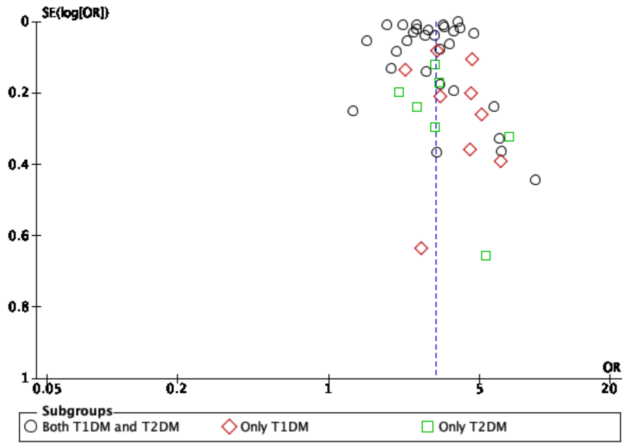

Supplementary Figure S4. Cesarean delivery

Funnel plots of comparison: PGDM vs Control

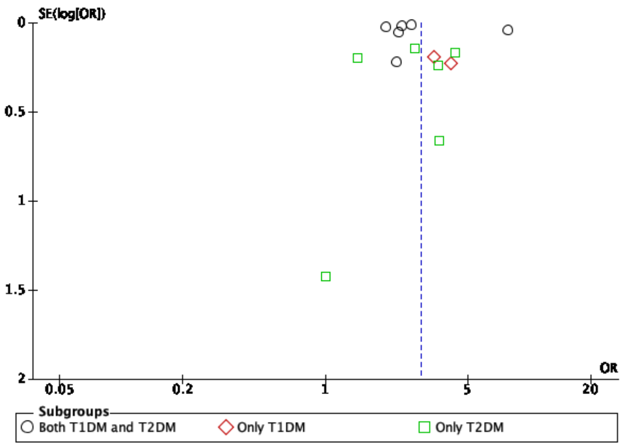

Supplementary Figure S5. Induction of labor

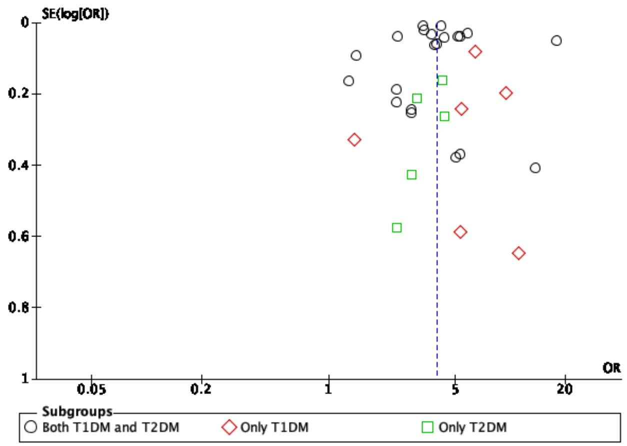

Supplementary Figure S7. LGA neonates

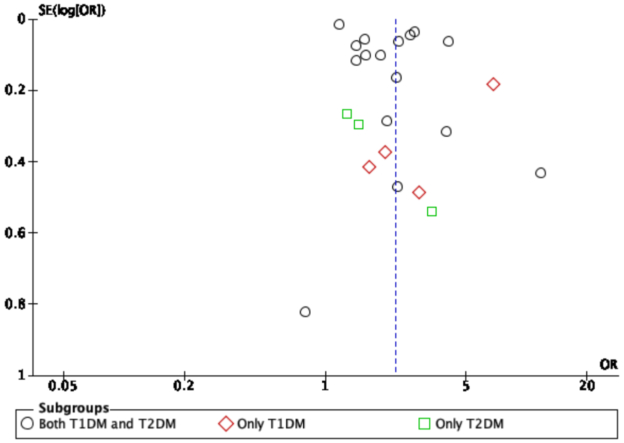

Supplementary Figure S6. Macrosomia

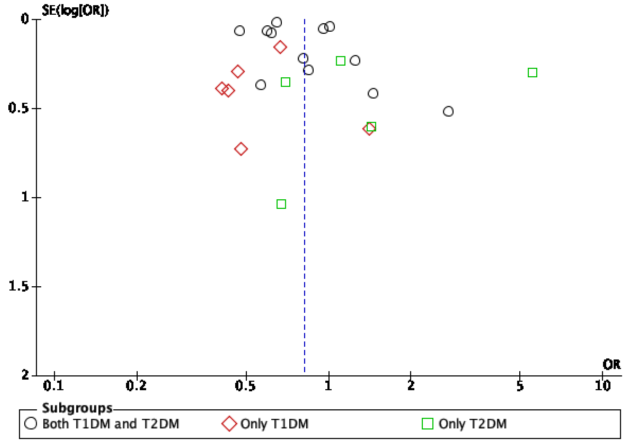

Supplementary Figure S8. SGA neonates

Funnel plots of comparison: PGDM vs Control

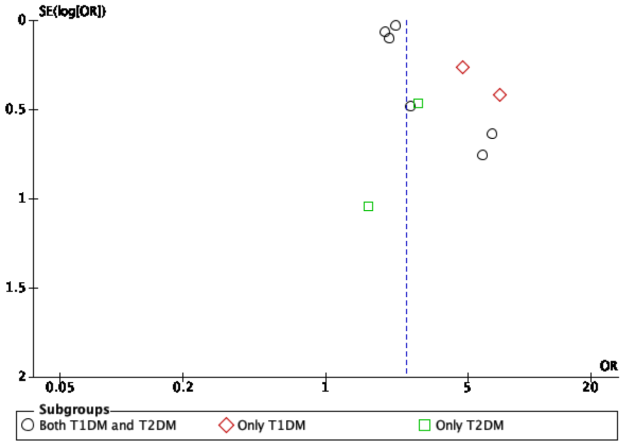

Supplementary Figure S9. Low Apgar score

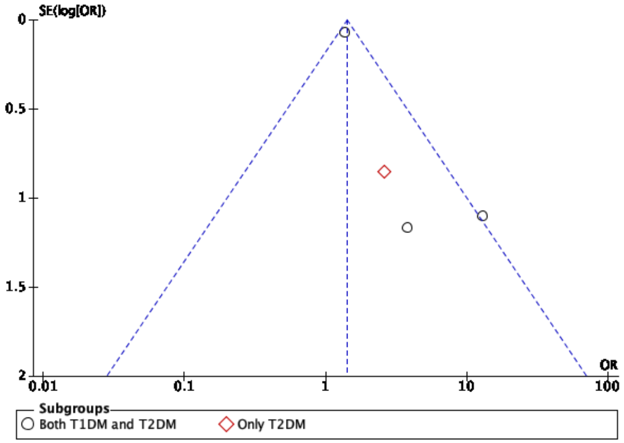

Supplementary Figure S11. Birth trauma

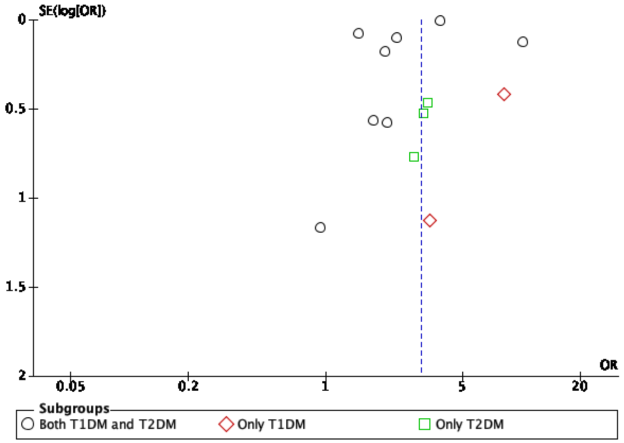

Supplementary Figure S10. Shoulder dystocia

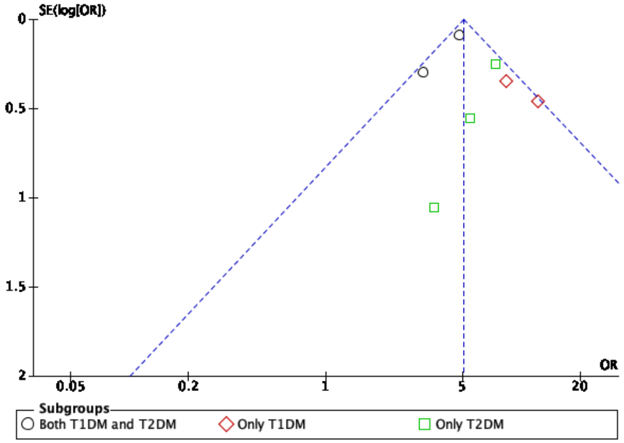

Supplementary Figure S12. Polyhydramnios

Funnel plots of comparison: PGDM vs Control

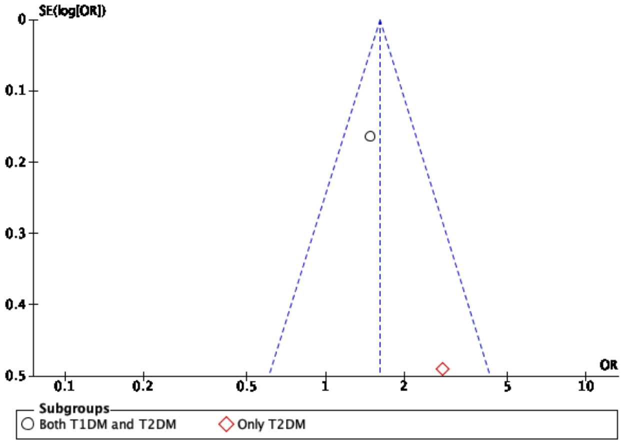

Supplementary Figure S13. Oligohydramnios

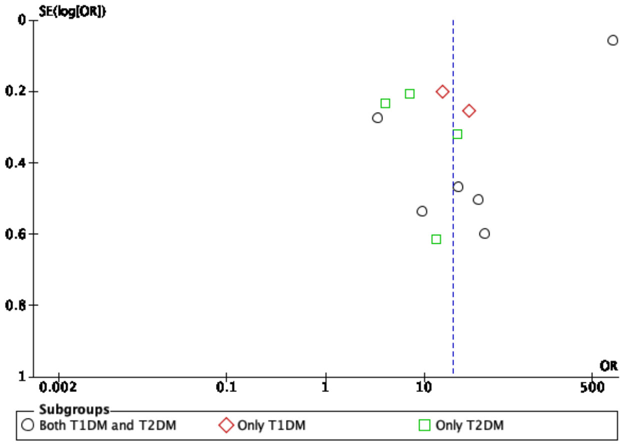

Supplementary Figure S15. Neonatal hypoglycemia

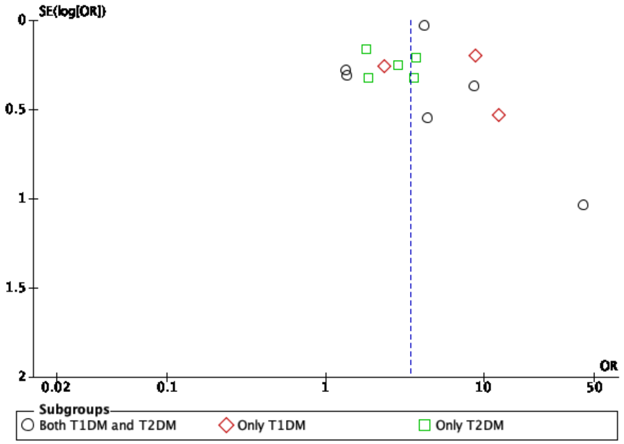

Supplementary Figure S14. Neonatal hyperbilirubinemia

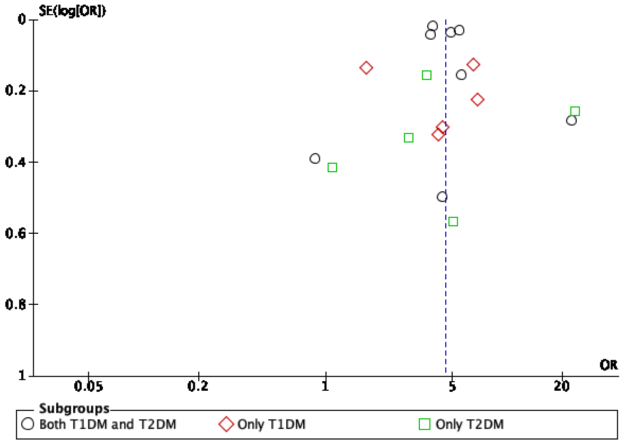

Supplementary Figure S16. NICU admission

Funnel plots of comparison: PGDM vs Control

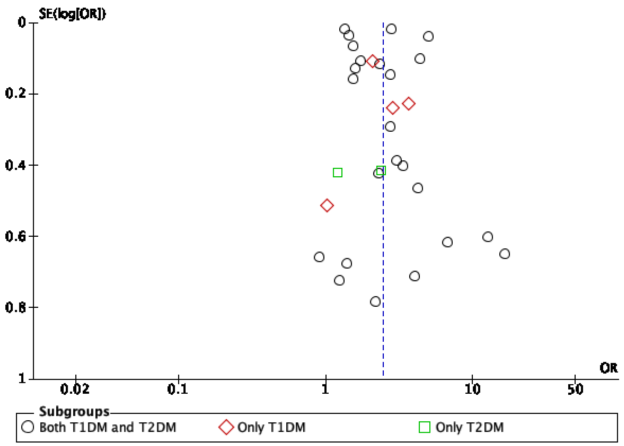

Supplementary Figure S17. Congenital malformations

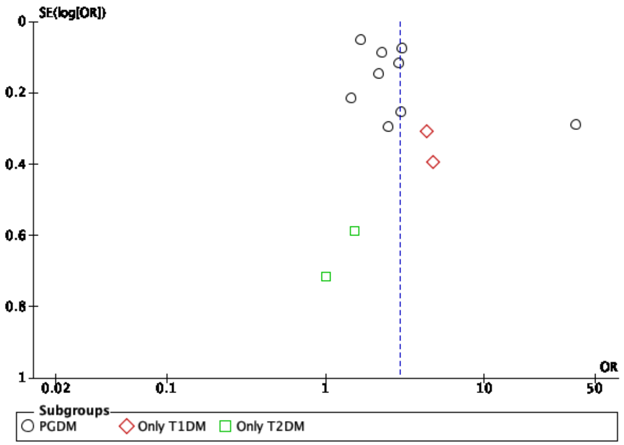

Supplementary Figure S19. Perinatal mortality

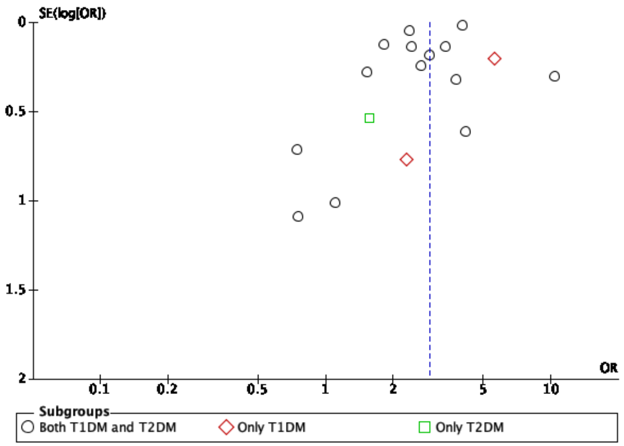

Supplementary Figure S18. Stillbirth
