# Supplementary material for: Pregestational Diabetes Mellitus and Adverse Perinatal Outcomes: A Systematic Review and Meta-Analysis
Source: J Clin Med. 2025 Jul 7;14(13):4789. doi: 10.3390/jcm14134789 (PMC12251443; doi:10.3390/jcm14134789)
Supplement: Supplementary file 1 [file jcm-14-04789-s001.zip › PGDM_supplementary/PGDM_Risk_of_bias_summary.pdf]

	SELECTION	COMPARABILITY	OUTCOME/EXPOSURE
Abell 2016	+	+	+
Abell 2017	+	+	+
Achkar 2015	?	?	?
Anderson 2012	?	+	+
Barakat 2010	+	+	?
Bashir 2019	?	+	?
Bashir 2019a	?	+	?
Battarbee 2020	+	+	+
Beyerlein 2018	+	+	+
Bicocca 2022	+	−	+
Billionnet 2017	+	?	+
Capobianco 2022	?	?	+
Chen 2023	+	?	+
Cynthia 2011	?	−	+
Dalfrà 2011	?	−	?
Di Lorenzo 2012	?	−	?
Dolk 2020	?	+	?
Eidem 2010	+	?	+
Fang 2023	+	?	?
Foeller 2015	+	?	+
Gardosi 2013	+	+	+
Giraldo-Grueso 2020	?	−	?
Gordon 2013	+	?	+
Gorsch 2023	+	?	+
Gortazar 2020	+	?	+
Gortazar 2021	+	?	+
Gualdani 2021	+	+	+
He 2023	+	+	+
Hunt 2012	+	+	+
Jang 2018	?	+	?
Jovanovi 2015	+	−	+
Kanda 2012	+	?	+
Kattini 2020	+	−	+
Kekki 2022	+	?	+
Knight 2012	?	−	?
Knight 2012a	+	+	+
Kohn 2019	+	?	+
KR 2010	?	−	+
Kuc 2011	?	?	?
Lai 2016	+	?	+
Lasheen 2014	?	−	+
Lemaitre 2023	+	?	+
Lin 2017	+	?	+
Lindsay 2003	?	?	+
Liu 2013	+	?	+
Lopez de Andres 2020	+	?	+
Loukovaara 2004	?	−	+
Luo 2022	+	−	+
Metcalfe 2017	+	?	+
Mirghani 2012	?	−	?
Morgan 2013	+	?	+
Ngwezi 2023	+	?	+
Owens 2015	?	+	?
Papageorghiou 2005	?	−	+
Par 2014	?	+	+
Patel 2015	+	?	+
Pereda 2020	+	+	+
Peticca 2009	+	?	+
Praprotnik 2021	?	−	?
Reddy 2010	?	+	+
Reitzle 2023	+	−	+
Riskin 2020	?	−	+
Schraw 2021	+	+	+
Seah 2021	+	−	+
Serehi 2015	?	+	?
Shefali 2006	+	?	?
Shour 2022	+	+	+
Son 2015	+	?	+
Stanton 2005	?	−	?
Stogianni 2019	+	?	+
Titmuss 2023	+	+	?
Wahabi 2012	+	?	+
Wei 2019	+	+	?
Wells 2015	?	−	+
Wright 2012	+	?	+
Xu 2014	+	?	+
Xu 2020	+	+	?
Yang 2019	+	+	+
Yanit 2012	+	?	+
Yves 2010	+	−	+
Zeki 2018	+	?	+
